# Bacterial Contamination of Angiographic Materials in Diagnostic and Interventional Neuroangiography

**DOI:** 10.1007/s00062-025-01526-3

**Published:** 2025-05-22

**Authors:** Christiane Franz, Claudia Fleu, Sophia Honecker, Manuela Schmiech, Dimah Hasan, Hani Ridwan, Omid Nikoubashman, Sebastian Lemmen, Martin Wiesmann

**Affiliations:** 1 Department of Diagnostic and Interventional Neuroradiology, Medical Faculty, RWTH Aachen University Hospital, Pauwelsstr. 30, 52074 Aachen, Germany; 2 Division of Infection Control and Infectious Diseases, Medical Faculty, RWTH Aachen University Hospital, Aachen, Germany; 3 Institut für Krankenhaushygiene und klinische Infektiologie, Colynshofstraße 57, 52074 Aachen, Germany

**Keywords:** Angiography, Bacteremia, Contamination, Intervention

## Abstract

**Purpose:**

Bacterial contamination has been reported to occur during angiographies, although data on its frequency and relevance are limited. The purpose of our study was to determine whether angiographic materials such as catheters and guide wires remain sterile during angiographies. We sought to differentiate between different materials, and to detect the frequency, the extent and the spectrum of bacterial contamination.

**Methods:**

We prospectively collected 698 fluid or material samples from 100 neuroangiographies. Per angiography we analyzed proximal ends and distal tips of catheters and guide wires, and fluid samples from the water container (working bowl) in which materials were stored during the angiography. We analyzed the frequency and extent of contamination and determined the bacterial spectrum.

**Results:**

The majority of samples (51.4%) were contaminated. There was no angiography that showed no contamination (0%). The highest proportion of contaminated samples was found in the fluid from the working bowl after completion of the examination (92.9%). Catheters and wires were contaminated in 34.1–49.2% of samples. Contamination of the samples increased with longer duration of the angiographic procedures. Most of the bacterial species were environmental or skin contaminants (86.2%).

**Conclusion:**

Bacterial contamination during diagnostic neuroangiographies or interventions is a frequent finding although its clinical significance is believed to be small. Bacterial contamination increases with longer duration of angiographic procedures.

## Introduction

Following the seminal work of Ignaz Semmelweis, Robert Koch, and Louis Pasteur in the late 19th century the implementation of infection control principles has become a cornerstone of modern medicine. Nevertheless, bacteremia frequently occurs after various medical procedures, such as urethral catheterization or dental procedures [1]. With regard to radiology, bacteremia has been reported to occur in 4–8% of angiographic procedures although it mainly remains asymptomatic [2–4]. So, these events seem to have little or even no clinical impact at least in immune competent patients, especially without valvular heart disease. According to current guidelines arterial angiographic procedures are not considered to constitute a significant risk for nosocomial infections. Therefore periinterventional antibiotics should not be administered, even in patients with heart valve replacements [5, 6]. However, little is known about immunocompromised patients or patients with intravascular devices. When immune response mechanisms fail, bacteremia becomes a bloodstream infection that can evolve into many clinical spectrums and is termed septicemia. Untreated and clinically significant bacteremia may progress to e.g. systemic inflammatory response syndrome (SIRS), sepsis, septic shock, or cerebral infection. Thus, infection control guidelines should be implemented locally to reduce the rate of preventable complications, and angiography hygiene protocols might need to be modified for high-risk patients. From a scientific point of view, neither the true incidence of bacteremia during neuroangiography nor the mechanisms leading to bacterial contamination are known. In a recent study, 25.3% of all fluid samples used in diagnostic and interventional neuroangiographies were contaminated with bacteria [7]. Moreover, it was recently published that 56.9% of samples from syringes used during neuroangiographies were bacterially contaminated [8]. To the best of our knowledge, bacterial contamination of catheters and wires used during neuroangiography has not been studied. We therefore performed an observational study to determine rates of bacterial contamination of catheters and wires used during typical diagnostic neuroangiographies and interventional procedures. In addition, we analyzed the contamination of fluids in which the catheters and wires were stored, and we performed a detailed analysis of all detected bacteria.

## Materials and Methods

### Sample Collection

After approval from the Ethics Committee of our institution (EK23/228), we prospectively collected samples during 100 neuroradiological catheter angiographies. These comprised 76 diagnostic neuroangiographies and 24 arterial neuroendovascular interventional procedures. None of our patients had a history of ongoing bacterial infection or was under antimicrobial treatment. Angiographies or interventional procedures were performed by a team of 7 neuroradiologists between January and May 2021. All examinations were performed in a dedicated neuroangiography operating room equipped with HEPA-filters according to national recommendations (DIN 1946-4). Patients’ groins were meticulously disinfected with antiseptics according to the national guidelines for infection prevention. Operators performed surgical hand disinfection up to the wrist with alcohol-based hand disinfectants, were dressed in a sterile single use surgical gown and wore a cap and a face mask at all times. All procedures were performed in accordance with the latest national recommendations for infection control and prevention [9] that were implemented in our hospital.

In a standardized approach, we examined neuroangiographic fluids (sterile NaCl 0.9% solution, Fresenius, Bad Homburg, Germany) from two bowls, which were placed on the angiography table and filled with NaCl fluid at the beginning of the angiography. One of the bowls was used to store catheters and wires during angiographies (working bowl). The other bowl was not touched between beginning and end of angiography (control bowl). From both bowls, 50 ml of fluid were drawn using sterile 50 ml syringes at the beginning and at the end of angiography, respectively. This resulted in 4 fluid samples per angiography.

Additionally, the diagnostic catheter and the probing wire used during the angiography were analysed. For this purpose, their distal tips (length 15–20 cm) were cut off with a sterile forceps and dropped into sterile containers. In this regard, “distal” corresponds to the part of the materials entering the blood stream of patients. Starting from the 37th of the 100 angiographic procedures we additionally analysed the proximal parts of catheters and wires, i.e. the part of the materials manipulated by the angiographer during the procedure. For this purpose, the proximal parts (length 15–20 cm) of catheters and wires were also cut off with a sterile forceps and dropped into sterile containers. This resulted in 2–4 samples of angiographic materials per angiography.

Individual samples that were obviously contaminated during collection or during processing in the laboratory were excluded from the analysis and discarded. Thus, a total of 698 usable fluid or material samples were obtained and included in the analysis.

### Microbiological Laboratory

Sample processing was performed under a laminar flow hood (Fisher Scientific, Schwerte, Germany) according to a laboratory standard protocol for processing sterile fluids. Fluid samples were directly filtrated under sterile conditions. Wires and catheter probes were each incubated in 500 ml sterile NaCl 0.9% solution and vortexed for 20 min on a shaker (Heidolph Instruments, Schwabach, Germany). Then, wire and catheter probes were removed with a sterile forceps and discarded. For sterile filtration, an aspiration system was loaded with sterile filters (0.2 µm, Sartorius, Goettingen, Germany) and funnels (Sartorius, Goettingen, Germany) and all samples were filtered separately. One sterile filter per fluid sample was then transferred to a microbiological culture medium (TSABA Agar, Thermo Fisher Diagnostics, Wesel, Germany). Incubation was performed for 48 h in an incubator (T = 37 °C). After incubation, colonies were counted and assessed macroscopically.

Microscopic assessment was performed by classical Gram staining. Subsequent species identification was performed using Matrix-assisted laser desorption ionization-time of flight mass spectrometry (MALDI-ToF MS) (Bruker Corporation, Billerica, MA, USA). If a bacterium was identified as Staphylococcus aureus, it was first subjected to MALDI-TOF analysis again.

If the MALDI-TOF analysis could not reliably rule out Staphylococcus aureus, a Pastorex™ Staph Plus Latex Agglutination Test (Bio-Rad Laboratories, Feldkirchen, Germany) was performed. If agglutination was positive, the isolate was applied to an MRSA plate (Brilliance™ MRSA 2 Agar, Thermo Fisher Scientific, Waltham MA, USA) and incubated as described above.

### Angiographic Procedures

Angiographies were performed by 7 neuroradiologists with 5 to 25 years of angiographic experience. Selection of angiographic materials and procedural details were left to the discretion of the angiographer. Prior to the start of the study, some investigators already used an incision film to tape the groin of the person to be examined before the puncture in order to cover the hole in the standard drape. Since it could not be ruled out that this might cause a reduction in bacterial contamination, the use of such an incision film was included in the analysis as an additional factor.

### Statistical Analyses

Statistical analysis was performed with SPSS 28 (IBM Statistics, Chicago Ilinois, USA). After testing for normal data distribution with Kolmogorow tests, t‑tests were applied. If data did not meet the requirements of normal distribution, chi-square tests or Mann-Whitney U tests were used as applicable. The Pearson correlation coefficient was calculated to investigate the relationship between variables. Results are reported as mean ± standard deviation, or median with inter-quartile range (IQR) if not normally distributed. All tests were two-sided. A *P* value < 0.05 was considered statistically significant.

## Results

A total of 698 fluid or material samples were included in the analysis as described above. Samples were obtained from 76 diagnostic angiographies and 24 interventional angiographic procedures. The number of colony-forming units (CFU) and the examination duration were not normally distributed (*p* < 0.05). The median duration for all angiographies was 1.38 h (IQR: 1.00 h; mean 1.81 ± 1.25 h). With a median duration of 3.50 h (IQR: 2.13 h; mean 3.43 ± 1.50 h) (*p* < 0.001) the interventional procedures were significantly longer than the diagnostic angiographies, whose median duration was 1.25 h (IQR: 0.75 h; mean 1.30 ± 0.53 h).

### Fequency and Extent of Contamination

Of the 698 samples evaluated, a total of 359 were contaminated (51.4%). There was no angiography that showed no contamination (0%). The frequency of contamination varied according to sample type (Table 1). The extent of contamination was determined based on the number of colony-forming units (CFU) per material sample or per 50 ml of fluid sample.

**Table 1:** Frequency of bacterial contamination according to sample type. Analysed samples comprise fluid samples from the working bowl, in which angiographic materials were stored, and a control bowl at the start (pre) and at the end (post) of the angiography, as well as samples of catheters and wires used during the angiographies

	Analysed samples (*n*)	Contaminated samples (*n*)	Rate of contaminated samples (%)
Working bowl (pre)	99	36	36.4
Control bowl (pre)	100	37	37.0
Working bowl (post)	99	92	93.0
Control bowl (post)	100	62	62.0
Catheter (distal tip only)	99	35	35.4
Catheter (proximal part only)	63	31	49.2
Wire (distal tip only)	62	23	37.1
Wire (proximal part only)	44	15	34.1
Wire (full length)	34	32	94.1

A total of 4281 CFU were counted on the 359 contaminated samples, which corresponds to an average of 11.9 ± 23.2 CFU per contaminated sample (median 4, IQR 8). The extent of contamination varied according to sample type (Table 2).

**Table 2:** Extent of bacterial contamination as determined based on the number of colony-forming units (CFU) according to sample type. Analysed samples comprise fluid samples from the working bowl, in which angiographic materials were stored, and a control bowl at the start (pre) and at the end (post) of the angiography, as well as samples of catheters and wires used during the angiographies

	Analysed samples (*n*)	Number of contaminated samples (*n*)	CFU total (*n*)	CFU (*n*) of all samples (mean ± SD)	CFU (*n*) of contaminated samples (mean ± SD)
Working bowl (pre)	99	36	213	2.2 ± 6.9	5.9 ± 10.5
Control bowl (pre)	100	37	173	1.7 ± 7.1	4.7 ± 11.2
Working bowl (post)	99	92	2353	23.8 ± 31.6	25.6 ± 32.1
Control bowl (post)	100	62	614	6.1 ± 18.1	9.9 ± 22.2
Catheter (distal tip only)	99	35	194	2.0 ± 5.1	5.5 ± 7.3
Catheter (proximal part only)	63	31	108	1.7 ± 3.4	3.5 ± 4.2
Wire (distal tip only)	62	23	272	4.4 ± 23.0	11.8 ± 37.1
Wire (proximal part only)	44	15	52	1.2 ± 2.3	3.5 ± 2.9
Wire (full length)	34	32	302	8.9 ± 12.2	9.4 ± 12.3

*CFU* colony-forming units; *SD* standard deviation

At the start of the angiography, the fluid samples from the control and working bowls did not differ significantly in the observed frequency of contamination (*p* = 0.926) or in the number of CFU (*p* = 0.944). The highest proportion of contaminated samples was found in the working bowl after completion of the examination. Contamination was detected in 92 of the 99 samples analysed (92.9%) and 23.8 CFU were found per sample on average (or 25.6 CFU per contaminated sample). In comparison, with 62.0% (62 out of 100 samples), the frequency of contamination was lower in the control bowl after completion of the examination (*p* < 0.001), and fewer CFU were found (6.14 CFU per sample or 9.90 CFU per contaminated sample, *p* < 0.001). The increases in frequency and extent of contamination between start and end of the angiography were statistically significant for both control bowl and working bowl, respectively (*p* < 0.001).

Almost all samples of angiographic wires were found to be contaminated, when the full length of the wires were analysed (32 of 34 samples, 94.1%). In comparison to that, when proximal parts and distal tips of wires and catheters were analysed separately, the frequencies of bacterial contamination were in a range of 34.1–49.2%, and thus significantly lower (*p* < 0.001, see Tables 1 and 2).

Although there was no significant difference in the frequency of contamination between distal wire tips and proximal wire ends (37.1% vs. 34.1%, *p* = 0.751), significantly more CFU were found on the wire tips (4.4 ± 23.0 vs. 1.2 ± 2.3, *p* = 0.017). There was also no significant difference in the frequency of contamination between catheter tips and wire tips (35.4% vs. 37.1%, *p* = 0.823), but significantly more CFUs were found on the wire tips (2.0 ± 5.1 vs. 4.4 ± 23.0,*p* = 0.003). There was no significant difference between distal catheter tips and proximal catheter ends in the frequency of contamination (*p* = 0.08) and the number of CFU (*p* = 0.155). There was no significant difference between catheter ends and wire ends in the frequency of contamination (*p* = 0.12) and the number of CFU (*p* = 0.228).

### Qualitative Analysis of Bacterial Spectrum

Qualitative analysis revealed a bacterial spectrum of 73 different types, which were grouped into 34 species (see Table 3). Of the 698 samples investigated, 359 showed bacterial growth: 142 (39.1%) of these showed monomicrobial contamination, 194 samples (53.4%) were contaminated with 2 to 5 different microorganisms, and 26 samples (7.5%) were contaminated with 6 to 10 different microbial species.

**Table 3:** Bacterial spectrum found in the samples using MALDI-ToF MS

Germ	Frequency (%)	Gram	Group
Acinetobacter spp.	0.64	negative	potentially pathogenic
Aerococcus viridans	0.32	negative	other gram-negative germs
Bacillus spp.	0.86	positive	environmental or skin germs
Brachybacterium muris	0.86	positive	environmental or skin germs
Brevibacillus spp.	0.11	positive	environmental or skin germs
Brevibacterium spp.	0.75	positive	environmental or skin germs
Brevundimonas diminuta	0.11	negative	other gram-negative germs
Cellulosimicrobium cellulans	0.11	positive	environmental or skin germs
Corynebacterium spp.	6.03	positive	environmental or skin germs
Cutibacterium avidum	0.11	positive	environmental or skin germs
Cryptococcus diffluens	0.11	yeasts	environmental or skin germs
Dermabacter hominis	0.86	positive	environmental or skin germs
Dermacoccus nishinomiyaensis	2.37	positive	environmental or skin germs
Kocuria spp.	6.03	positive	environmental or skin germs
Kytococcus sedentarius	0.54	positive	environmental or skin germs
Microbacterium testaceum	0.11	positive	environmental or skin germs
Micrococcus spp.	18.3	positive	environmental or skin germs
Moraxella spp.	7.97	negative	other gram-negative germs
Neisseria cinerea	0.11	negative	other gram-negative germs
Pantoea septica	0.11	negative	other gram-negative germs
Paracoccus yeei	0.75	negative	other gram-negative germs
Pseudarthrobacter sulfonivorans	0.22	positive	environmental or skin germs
Pseudoclavibacter spp.	0.11	positive	environmental or skin germs
Pseudoglutamicibacter cumminsii	0.11	positive	environmental or skin germs
Psychrobacter sanguinis	0.22	negative	other gram-negative germs
Rhizobium radiobacter	0.22	negative	other gram-negative germs
Rothia dentocariosa	0.22	positive	environmental or skin germs
Mold fungus	0.11	molds	environmental or skin germs
Staphylococcus aureus	1.72	positive	potentially pathogenic
Staphylococcus lugdunensis	1.61	positive	potentially pathogenic
Staphylococcus spp. (coagulase negative)	43.92	positive	environmental or skin germs
Streptococcus dysgalactiae (non-hemolytic)	0.11	positive	environmental or skin germs
Streptococcus spp. [hemolytic]	0.11	positive	environmental or skin germs
Other	4.2	pos./neg.	environmental or skin germs

Most of the bacterial species were environmental or skin contaminants, which caused 86.2% of CFU. However, three groups of potentially pathogenic bacteria were found: (1) *Staphylococcus aureus* and *Staphylococcus lugdunensis* (3.3% of CFU), (2) *Acinetobacter spp.* (0.65% of CFU), and (3) other gram-negative bacteria (9.8% of CFU).

The three most common bacteria in terms of angiographic examinations and samples in which they were found were *Micrococcus luteus* (in 82 angiographies and 165 samples), followed by *Staphylococcus epidermidis* (in 68 angiographies and 106 samples) and *Staphylococcus hominis* (in 61 angiographies and 98 samples). The highest number of colony-forming units were found in *Staphylococcus epidermidis* (904 CFU) followed by *Staphylococcus capitis* (578 CFU) and *Staphylococcus hominis* (517 CFU). Environmental or skin bacteria were detected in each angiography (100%).

### Factors Influencing the Contamination of the Samples

#### Type of Angiography

Whether the angiography was a diagnostic angiography, or an interventional procedure had no significant influence on the frequency of contamination or the number of CFU/procedure found (*p* > 0.05).

#### Duration of the Angiography

Median duration of the angiographic studies was 90 min. We therefore compared all angiographies lasting < 90 min (*n* = 50) with the angiographies lasting ≥ 90 min (*n* = 50). When the duration of the angiographies was < 90 min, 48.8% of samples were contaminated and 32.5 CFU/procedure were detected on average. In comparison, when the duration of the angiographies was ≥ 90 min, 56.5% of samples were contaminated and 53.1 CFU/procedure were detected on average. This difference was statistically significant for both frequency (*p* = 0.048) and extent of contamination (*p* = 0.001).

#### Use of an Incision Film for Groin Puncture

In 20 angiographic procedures an incision film was used for groin puncture. In these cases, 50.5% of samples were contaminated, and 33.8 CFU/procedure were found on average. In the remaining 80 procedures, in which no incision film was used for groin puncture, 53.6% of samples were contaminated, and 46.7 CFU/procedure were found on average. The reduced frequency of contamination, when an incision film was used, was statistically significant (*p* = 0.037). However, the difference in the extent of contamination was not found to be statistically significant (*p* = 0.208).

## Discussion

During angiographies materials such as catheters and guide wires are inserted into the bloodstream of patients. The purpose of our study was therefore to determine whether angiographic materials remain sterile during these procedures. To the best of our knowledge, our study is the first to investigate bacterial contamination of angiographic fluids, catheters and wires during neuroangiographies.

The main finding of our study was that at the end of angiographies the majority of fluids and material samples were contaminated (51.4%), and that there was not a single angiography or intervention which showed no contamination, although all angiographies had been performed under routine sterile conditions.

In previous studies, bacteremia has been reported to occur in 4–8% of angiographic procedures, although typically asymptomatic [2–4]. In a series of 100 angiographies Shawker et al. found a contamination of catheters or wires in 23% of cases, and transient bacteremia in 4% of cases [3]. Sande et al. studied 106 patients undergoing cardiac angiography. All venous blood samples drawn during the angiography or within 24 h after angiography were sterile. However, 8% of blood samples drawn from the angiography catheters during the procedure were contaminated [2]. After dental extraction bacteremia has even been found in 100% of cases [1]. Usually this does not have any clinical consequences as long as patients are immune competent. Nevertheless, bacterial contamination should always be a matter of concern in clinical medicine. This also holds true for the field of neuroradiology. Consequently, all infection control standards need to be constantly re-evaluated in hospital routine.

Neuroangiographies have not been the focus of attention with respect to bacterial contamination and have been reported to be associated with low complication rates [10]. However, potential non-microbial contamination has already been reported [11]. Concerning bacterial contamination, Tress et al. [12] found a contamination in 3 out of 7 patients who underwent angiography. In two other neuroangiographic studies, contamination of sterile liquids was found in 25.3% [7] and syringes were contaminated in 56.9% [8].

Our knowledge of the sources of bacterial contamination is limited. Our study was not designed to analyze this. In addition, the efficacy of measures to prevent transmission is limited. Standard angio suites do not meet operating room infection control standards concerning air flow and patient/personnel-access regulations. Therefore, bacteria are to be expected to be present in the air when angiographies are performed under routine sterile conditions. Thus, airborne transmission is conceivable. Specifically, both the working bowl and the control bowl were exposed to filtered but not “sterile” air during the angiography. Consequently, although the control bowl was not touched between beginning and end of the angiography, contamination through airborne transmission is conceivable, and this may explain the findings of contamination in the samples from the control bowl. On the contrary, Kabbasch et al. considered airborne transmission as unlikely since intermittent coverage of the fluid bowls did not lead to a reduction of contamination in their study [7]. Our results also speak against airborne transmission as a relevant factor for contamination. At the end of angiography, fluid samples from the working bowl were significantly more contaminated than fluid samples from a control bowl placed on the angiography table, although both bowls were similarly exposed to the air.

Although sterile surgical gloves were used initially, it is well known that these can become heavily contaminated over time during procedures due to multiple contact with patient skin, material and different surfaces; thus bacterial contact transmission via gloves seems to be feasible. Consequently, the fluid in the working bowl cannot be considered to remain sterile after multiple usage during the same procedure. Likewise, high rates of contamination could therefore be expected for materials often touched by the operators. This hypothesis compares well with the results of a previous study, in which nearly all syringes used during neuroangiographies were contaminated on their outside (91.0%) [8]. In our study, samples from different parts of catheters and wires were found to be contaminated in 34–49% of cases. It is possible that contact with the gloves of the operators caused at least a certain part of contamination of angiographic materials and the fluid in the working bowl.

In accordance with previous studies, our data show that contamination increases with duration of the angiography [8]. This is understandable and should hold true both for airborne and contact transmission. In our opinion, the increased frequency of contact with the operators’s gloves may here play a pivotal role.

Despite the purely observational design of our study and its small sample size we consider it interesting that our results indicate that using an incision film for groin puncture may be beneficial to reduce bacterial contamination. Of course, patients’ groins were disinfected as prescribed by standard hygiene protocols. Nevertheless, this does not mean that the disinfected skin is or will remain sterile. Subsequent contact with the operators’ gloves may therefore constitute a possible source for contamination. As reported above, 50.5% of samples were contaminated, and 33.8 CFU/procedure were found on average when an incision film was used. In contrast, when no incision film was used, 53.6% of samples were contaminated (*p* = 0.037), and 46.7 CFU/procedure were found on average (*p* = 0.208). However, these results should be confirmed in a randomized study design with a larger cohort of patients.

Healthy human skin is densely colonized by a variety of microorganisms, such as bacteria and fungi, which are collectively referred to as skin flora. They are not pathogenic, and help to protect humans from pathogens. The skin flora is therefore now also referred to as skin microbiome. All surfaces of the environment in which we live are also colonized by a variety of microorganisms. The majority of these microorganisms, which are also found in the air we breathe, are non-pathogenic for humans as well, and are referred to as environmental germs.

The majority of bacterial species detected were non-pathogenic environmental or skin contaminants (86.2%). For immune competent patients these will most probably not have any consequences but can be cleared due to the bactericidal activity of the blood. To put this into context, even brushing teeth caused relevant bacteremia in 23% of cases [13]. However, pathogenic bacteria, like *Staphylococcus aureus *and *Staphylococcus lugdunensis* were also detected.

It is a limitation of our study, that patients were not tested for bacteremia by blood cultures. In addition the follow-up period was too short to definitely rule out any clinical effects. With 23% of distal wire tips and 35% of distal catheter tips having been contaminated it is quite likely that bacteremia was caused during angiography in some cases. Nevertheless, our study study design does not allow conclusions on the clinical significance of our microbiological findings.

In the context of angiographic procedures, our knowledge on preventive strategies to reduce bacterial contamination is limited. Reducing the extent of airborne bacterial transmission would probably mandate to perform angiographic procedures under operating room infection control standards concerning air flow and patient/personnel-access regulations. Strategies which could be tested to reduce transmission through contact with the operators’ gloves include changing gloves after groin puncture, changing gloves after prescribed time intervals (e.g., every two hours), or wearing two pairs of gloves. To the best of our knowledge, optimized strategies for material handling and storage with regard to bacterial contamination have not been proposed.

To the best of our knowledge, our study is the first to report on bacterial contamination of angiographic fluids, catheters and wires during neuroangiographies. Angiographically induced bacteremia seems to be clinically irrelevant in a standard patient population. Still, the frequency and extent of bacterial contamination during angiography must be addressed and aimed to be reduced, especially with regard to critically ill or immunocompromised patients. Thus, the results presented in this study can serve as a starting point for future studies concerning the efficacy of potential mechanisms to reduce bacterial contamination. It was not the aim of our study to investigate different routes of bacterial transmission. With respect to the lack of data concerning primary transmission routes of bacteria, at this point there is ample room for speculation, and further studies are needed to focus on this issue and consequently decrease the risk of transmission.

## Limitations

Limitations of our study are that this was a descriptive study only and we did not vary angiographic conditions to test for potential effects on bacterial contamination. We also did not investigate actual bacteremia in our patients, e.g. obtaining blood cultures, nor did we add to clinical routine to detect delayed complications secondary to bacteremia such as secondary fever or pneumonia. A further limitaion is the limited samples size of our study.

## Conclusions

Bacterial contamination during diagnostic neuroangiographies or interventions seems to be a frequent finding although its clinical significance seems to be small. Bacterial contamination increased with longer duration of angiographic procedures. Most of the bacterial species detected were environmental or skin contaminants although pathogenic bacteria were also found in a minority of samples. Repetitive contact of angiographic materials with gloves is the most feasible route of transmission, however this needs to be confirmed in future studies.

## Data Availability

The raw data supporting the conclusions of this article will be made available by the authors on reasonable request.
